# “It's not just a matter of getting technology into buses”: An activity-centred approach to bus drivers’ work sustainability in an age of automation

**DOI:** 10.1177/10519815261445418

**Published:** 2026-05-12

**Authors:** Daniel Silva, Liliana Cunha

**Affiliations:** 1 Faculty of Education and Psychology at the Catholic University of Portugal, Porto, Portugal; 2 Center for Psychology at University of Porto (CPUP), Porto, Portugal; 3 Faculty of Psychology and Education Sciences of the University of Porto, Porto, Portugal

**Keywords:** public passenger transport, automation, automated buses, working conditions, occupational health, just transition, qualitative research

## Abstract

**Background:**

In the context of current challenges facing public passenger transport, automation is presented as a leading solution for achieving more efficient, safer, and more sustainable mobility. This is reflected in the growing number of pilot projects and trials with automated buses across Europe. Yet, debates on automated public transport are often dominated by either technocentric perspectives or concerns about passenger acceptance.

**Objective:**

This study adopts a perspective that has been insufficiently considered in research on automated public transport: the viewpoint of bus drivers. This neglect has meant that drivers’ work experience and their knowledge of the issues affecting public transport remain residual.

**Methods:**

A qualitative research design was employed, involving bus drivers from a public transport company in a metropolitan area of Portugal. Discussion sessions were organised as spaces for dialogue and reflection on work activity and its potential evolution with automation.

**Results:**

Four main themes emerged, illustrating the constraints that shape drivers’ current work and how these have contributed to the deterioration of job quality. Building on this, the drivers projected their probable future work with automated buses, showing that this transition intersects with much more than technology alone. It requires attention to working conditions, occupational risks, bus scheduling, and even urban planning.

**Conclusions:**

As investment in automated public transport grows, promising to redefine drivers’ work, our results help identify key organisational and policy factors that need to be addressed to promote sustainable work and secure a worker-led just transition to automation in public transport for bus drivers.

## Introduction

Public Passenger Transport (PPT) plays a crucial role in ensuring mobility and access to a wide range of activities, goods, and services. Within the European Union (EU), road transport represents a significant component of PPT, with bus transport accounting for 7.4% of total passenger-kilometres.^1^ In urban contexts, however, buses hold a higher modal share due to the shorter and more frequent nature of journeys within metropolitan areas.^2,3^ As such, buses are key to Urban Public Passenger Transport (UPPT), contributing to more inclusive access to cities. The importance of bus transport is also reflected in its social dimension. Across the EU, the industry is estimated to employ around 1.683 million people,^1^ including 900,000 bus drivers.^4^

These figures underscore the economic and social relevance of bus transport and help explain the continued policy attention it has received. Improving the efficiency, safety, and sustainability of urban bus systems has long been central to European transport policy and has been recently reaffirmed through the European Green Deal framework,^5,6^ which addresses major challenges linked to transport externalities, including greenhouse gas emissions, pollution, congestion, and road safety.^6^ Within this policy context, UPPT systems are increasingly encouraged to harness technological advances in automation, digitalisation, and decarbonisation, particularly through the introduction of automated vehicles within the “mobility of the future” paradigm.^7^

Against this background, the present study examines the work activity of bus drivers and their perspectives on the potential introduction of Automated Buses (ABs) into UPPT systems. By adopting an activity-centred perspective rooted in activity ergonomics and work psychology, the study explores how drivers’ experience can inform the design and implementation of automated public transport systems. In doing so, the article contributes to ongoing debates on sustainable work and on the need to ensure a just transition to automation for bus drivers.

### Two prevailing approaches to improving UPPT

When planning future UPPT systems, two broad approaches stand out. The first, a “techno-centric approach”, focuses on technological innovations. The second, a “citizen-centred approach”,^8^ also termed “sociocentrism”,^9^ explores how public transport can better meet passengers’ needs, particularly in terms of the availability of AB services and accessibility to mobility.^9,10^ This approach also examines passengers’ attitudes towards automated public transport and public acceptance of ABs.

While both approaches are valuable and necessary, their predominance tends to shift attention away from the workers who ensure the delivery of transport services^13–16^ and the role they may play in implementing ABs in future UPPT systems intended to be more efficient and sustainable. In this context, bus drivers are often relegated to a “backseat role” in discussions on vehicle automation.^14^ Likewise, Vitrano and Kębłowski^15^ note that research on future transport sustainability tends to prioritise “improving service efficiency and quality to reflect diverse needs of passengers and attract them to public transport” (p. 397), while overlooking the implications of such technological innovations for workers. And when these impacts are considered, the debate typically focuses on quantifying potential job losses from techno-deterministic perspectives,^12^ disregarding the organisational contexts of bus drivers’ work, as well as the experiential know-how they develop to ensure passenger safety, comfort, and service reliability.^13,14,18,19^ Consequently, bus drivers may be expected to accept and adapt to automated systems once these are implemented, bridging the gap between idealised system design and the complexities of urban environments that often exceed automation capabilities.^11,13^ This raises the risk of positioning drivers as a “variable of adjustment” in a system that neither recognises the complexity of their work nor draws on their expertise in shaping AB integration.

### Problem statement and objectives

This article addresses the research gap that keeps professional drivers in a peripheral position in discussions on future UPPT systems with ABs. Extending our work presented at the 22^nd^ Triennial Congress of the International Ergonomics Association,^11^ this study focuses on bus drivers’ current work. An activity-centred perspective is adopted, rooted in the fieldwork traditions of activity ergonomics and work psychology.^21–24^ This “intrinsic approach” to work design^23,25^ contrasts with top-down perspectives that take professional activity as a residual component in techno-organisational transitions.^26^ Accordingly, the exploration of future activity with ABs, and the underlying conditions required for more sustainable public transport, is grounded in the current work of bus drivers, identifying the constraints that characterise their work situations and the know-how developed in response. Through the collective discussion of this expertise, it is possible to reveal both the effectiveness of professional knowledge and its potential to improve working conditions.^24^

This approach also differs from perspectives that address the introduction of new technologies mainly through individual acceptability, based on attitudes towards technology. According to an activity-centred perspective, it is not the technology itself that is considered acceptable or not^27,28^; instead, the focus is directed towards the context in which the new technology will be used, so that its acceptance is considered in a contextualised manner, taking into account its possible effects on how work is performed. The goal, therefore, is not to specify the technical features of a new work system, but to integrate the new technology into the workplace in ways that enable a favourable reconfiguration of work practices by supporting workers’ skills and improving occupational health and well-being.^13^

Following a qualitative research design with a group of bus drivers from a metropolitan PPT company in Portugal, we sought to understand their work activity, constraints, and perspectives on future activity with ABs. This approach preserves the potential for intervention based on what professional experience reveals as most significant for the successful integration of ABs. The following questions guided the research:

- What conditions and key factors are required to ensure a more efficient and sustainable public transport system in the context of AB adoption?
- How do bus drivers prospect their future work activity, considering their skills and well-being?

In tandem with the contributions of activity ergonomics and work psychology in supporting design projects,^20,21,25,29^ this approach aims to ensure that workers can take ownership of the technological solutions and the associated transformations of their work, both before these changes are deployed and throughout implementation.

## Research underpinnings

### The work of bus drivers: on the brink of unsustainability?

When considering the sustainability of public transport and its advancement through automation technologies, financial and environmental dimensions often take centre stage. In doing so, another critical dimension tends to be overlooked: the social dimension of PPT sustainability,^15,30–32^ namely, bus drivers themselves. As Remy and Guseva Canu^31^ note, this oversight persists despite the bus driving profession having long been recognised as one of the most health-impaired worldwide.

The very demanding working conditions to which bus drivers are exposed and their effects on drivers’ declining well-being have been consistently documented.^15,30,31,33–35^ Frequently reported issues include long working hours and irregular schedules, prolonged static driving positions, lack of sanitary and rest infrastructures, or exposure to tight deadlines and intensified pace-related demands. Low wages, as reported by the European Transport Workers’ Federation,^33^ further exacerbate this reality, opening the door to pressures to continue driving (e.g., shortening breaks, working overtime) despite the fatigue drivers feel.

Recent studies in Portugal corroborate this scenario,^30,35^ showing that high-pressure working environments interact with the deterioration of bus drivers’ perceived health. Silva et al.'s^35^ study revealed a high prevalence of severe musculoskeletal symptoms, along with elevated anxiety and irritability, which may be more pronounced among bus drivers working in areas with heavier traffic congestion. In fact, for metropolitan bus drivers, these constraints are even stronger, inasmuch as they have to cope with higher traffic density, more unexpected events on urban routes, and situations of tension or hostility with the public.^13,14,36^

Such conditions, coupled with declining perceptions of job quality, have fuelled an exodus from the profession. Growing labour shortages and retention difficulties are now recognised as the Achilles’ heel of PPT across Europe.^4,32,37^ The sector struggles to attract new drivers who view the work as unappealing and unhealthy, whilst the current workforce no longer wants to do the job under such harsh working conditions, leading many to either leave the profession or express a desire to retire before the statutory age. This trend is likely to intensify: the International Road Transport Union^4^ estimates that the bus driving profession's lack of attractiveness currently results in around 105,000 unfilled positions in Europe, potentially rising to 275,000 by 2028 if working conditions remain unchanged.

### Automation to reconfigure working conditions and raise the attractiveness of the job

Faced with an industry that appears to be in crisis, there is growing investment in automated driving applied to PPT. Although concerns over potentially disruptive effects on employment remain,^32,37,38^ the transition to automated public transport is viewed as an opportunity to enhance job quality and tackle workforce shortages. As stated by the European Commission,^37^ “automation and digitalisation are expected to improve working conditions, offering workers more flexible working hours and higher safety levels, and removing many monotonous and physically difficult tasks” (p. 24).

In the EU context, recent years have seen a multiplication of projects and pilots aimed at developing and implementing automated shuttles and full-size ABs within UPPT systems. Whilst automated shuttles have been deployed in many European cities,^39,40^ full-size bus automation has progressed more slowly due to additional challenges posed by their length and capacity. According to data from the CAD – Knowledge Base on Connected and Automated Driving,^41^ 88 research and innovation projects and demonstrations involving ABs are currently underway or have been completed across Europe.

This investment in vehicle automation is accompanied by a reconfiguration of work. It is worth noting that automated driving does not equate to driver replacement at the wheel. Automated driving is usually classified into five levels of automation, with levels 1–4 requiring human intervention^a^.^42^ Most European trials have operated under conditional automation (level 3), where drivers must still be prepared to resume control when requested.^40^ Nevertheless, reduced reliance on manual and physical work may render the profession more attractive.^38^ Also, automated driving is expected to require different skill sets (e.g., IT- and supervision-related skills), opening possibilities for upskilling and potentially higher wage levels.^37^ Together, these transformations are expected to increase the profession's attractiveness: they may help current bus drivers remain in the job longer, while also making the profession more appealing to younger drivers^37,38^ – an especially pressing need given an ageing workforce, with an estimated 32% of European bus drivers aged 55+.^4^

Although the social dimension, centred on bus drivers’ work and occupational health and safety, has often remained subordinate, increased vehicle automation is expected to provide a pathway to reconciling the three pillars of PPT sustainability: economic, environmental, and social.

## Methods

### Research context

The study was conducted with bus drivers from a company providing PPT services in one of Portugal's main metropolitan areas. This company had participated in a European project focusing on integrating AB fleets into UPPT across six partner cities. Although the Portuguese pilot planned for 2020 was cancelled due to COVID-19, the company participated in the project's remaining activities (e.g., route planning, technical infrastructure). This means that, whilst its bus drivers did not gain direct work experience with ABs, driving automation and AB integration were on the company's agenda. Therefore, this context offered an opportunity to explore drivers’ perspectives on how ABs might improve UPPT systems and the potential implications for their work. According to an activity-centred approach to work design,^21,23,29^ while future work activity cannot be observed directly, current activity can nonetheless provide valuable insights. Thus, the focus is on exploring “current situations whose analysis makes it possible to clarify the objectives and conditions of future activity”.^21 (p.364)^

Bearing this in mind, we contacted the company and its Works Council to present the study and its objectives. Following approval, bus drivers were recruited through announcements disseminated by the Works Council. Ten male drivers agreed to participate, with an average age of 47 years and an average seniority in UPPT of 21.3 years. All participants held permanent contracts as “public service drivers” and operated on intra-urban routes shorter than 50 km. Regarding the buses currently operated, they are equipped with automatic gearboxes and 360-degree camera technology – an assisted driving feature that automatically activates at low speeds, providing drivers with a complete real-time view around the bus and reducing blind spots.

### Survey design and data collection

For data collection, we conducted three collective sessions with the bus drivers, conceived as spaces for dialogue and reflection on work activity.^43–45^ The study design thus assumed an intrinsically dialogical nature, in which collective discussion serves as a resource for considering and debating the reconfiguration of working conditions with automation technologies from the perspective of those who actually perform the work. From this standpoint, as Cippelletti et al.^43^ observe, the design of technology is not an end in itself but rather “a pretext, a means, for (re)establishing collective reflection and spaces for professional dialogue” (p. 251) aimed at improving existing situations and reflecting on technologies that could be integrated into them.

Throughout the three collective sessions, the discussion was structured to progressively approach future activity whilst taking the current work reality as the starting point. This strategy can be situated within the methodology of eliciting activity.^45^ The sessions were thematically structured as follows (see Table 1). The first two sessions focused on eliciting the current work activity in UPPT, spatially, temporally, and contextually. The elicited activity then served as the basis for the prospective exercise in the third session, where the drivers envisaged their probable future activity with ABs and discussed increasing levels of automation. To support collective reflection in this session, a short video was shown, outlining the operation of an AB and the actions required from workers^b^. This video depicted the operation of an AB along a 2.2 km urban route, involving interactions with other road users (pedestrians, cyclists, and other vehicles), stops at bus stops, and yielding manoeuvres (e.g., at pedestrian crossings and intersections). It is important to note, however, that the video presented a relatively “ideal” urban operating scenario, insofar as the road environment met many of the conditions favourable to automated vehicle operations, such as clear road markings, the absence of surface irregularities, and no illegal parking obstructing the AB's route.

**Table 1.** Collective sessions with the bus drivers: objectives and example questions.^13^

	Session 1	Session 2	Session 3
Objective	- Characterisation of current activity, considering its conditions and work organisation	- Identification of occupational risks and their perceived impacts on health and job attractiveness - Exploration of conditions under which automation may address current difficulties	- Identification of conditions for more sustainable and efficient UPPT - Prospective exercise on probable future activity^21^ supported by video mediation
Examples of questions	- How are bus routes and work schedules allocated? - What are the main requirements for being a bus driver in this city? What skills are essential? - What is not known about bus drivers’ work? How do you think the activity is viewed by outsiders?	- What are the most difficult work situations to handle? What factors influence them? - Could you describe one of these situations and the strategies to deal with it? - What are the risks you face? How do these manifest in your health and well-being? - How do you think automation could address these difficulties?	- What factors do you think should be discussed as the transition to automated transport is being considered? - How do you view your future activity in this scenario?* - What could be more difficult when working with ABs?* - What do you think will happen to the skills associated with conventional driving?*

*Examples of questions posed after viewing the video.

The video was not intended to shape the bus drivers’ views in advance; rather, it served as a means to elicit activity and act as a mediator of reflection. Building on the analysis of current activity carried out in the first two sessions, the video mediation also aimed to strengthen the verisimilitude of activity descriptions. In Van Belleghem's^45^ words, “here, present activity is a *pre-creation* of future activity” (p. 293).

All the sessions took place at the company's facilities between January and February 2023, lasting 90–120 min. All the bus drivers (*n* = 10) participated in the three sessions, which were audio-recorded and subsequently transcribed using NVivo software (v14), obtaining a verbatim transcript of each session. Prior to data collection, ethical approval for this study was obtained from the Ethics Committee of the FPCEUP (approval number: 2021/03-11b).

### Data analysis

The analysis was structured into two main stages, following a reflexive thematic approach^46,47^ to identify, analyse, and describe themes within qualitative data. This inductive, data-driven approach enabled the categorisation of the discussions into emerging themes, providing a detailed and interpretative account of the data.

We first conducted a reading aimed at familiarising ourselves with the dataset and generating an initial narrative (in vivo codes) from each discussion session. The participants’ own words and terminology were used to generate the initial codes. At the same time, it was necessary to go “beyond the data”, making interpretations where relevant to the research questions, while preserving the latent meaning conveyed by the participants.^46^ This means that following an inductive thematic analysis does not imply that the method is atheoretical. As Braun and Clarke^46^ clarify, despite not having a theory that predetermines the coding process (i.e., themes do not preexist the analysis), “thematic analysis can never be conducted in a theoretical vacuum; researchers always make assumptions about what data represent (…), what can be claimed on the basis of these data, and indeed what constitutes meaningful knowledge” (p. 337).

Moving into the second, more systematised coding stage, an initial set of themes was surfaced from the codes, reflecting their salience and relevance to the research questions. We followed a collaborative approach consistent with the principles of thematic analysis. The first author performed initial inductive coding, followed by three collaborative rounds between the two authors to critically review, refine, and merge codes, based on areas of similarity and overlap. Where necessary, code and theme formulations were adjusted to better reflect the participants’ meanings and to incorporate our interpretations, grounded in work psychology and activity ergonomics. This process enabled us to reach the final list of themes and subthemes synthesising the bus drivers’ viewpoints. In total, this analytical process generated 19 initial codes, which were iteratively developed into four final themes and nine subthemes. Illustrative data extracts are presented in Section 4.

## Results and discussion

The analytical structure of the four main themes, their corresponding subthemes, and illustrative quotes is presented in Table 2.

**Table 2.** Themes, subthemes and examples of supportive quotes.^13^

Theme	Subtheme	Bus driver	Quotation
It is not just about driving	Transporting people	42-year-old bus driver, with 21 years of seniority	*“Especially when someone with reduced mobility gets on. If there's a seat free, I let them sit down first. Otherwise, I wait until I see they’re steady, then I pull off gently, to make sure they don’t fall. But honestly, whether the bus is packed or nearly empty, you always feel like there's someone behind you. **You’re never just driving, you’re carrying people**”.*
	Knowing the city by heart	53-year-old bus driver, with 26 years of seniority	*“I know where the potholes are, where there’re traffic jams, delays, everything. It's not just about knowing the bus route, it's about knowing at what time it causes problems, where we’ll fall behind schedule, what detours are possible if a street is closed, how to reroute. I mean, **after all of these years, the city is in our heads**”.*
A job no one wants anymore	Always running late	47-year-old bus driver, with 13 years of seniority	“(…) *the route schedules are impossible to meet. So, we start already upset, because we know we’ll be running late, and this will lead to conflicts with the passengers waiting at the bus stops. It*'*s an immediate source of tension. **There’re mornings when I don’t even get up from the seat**. We don’t have time to go to the bathroom, and if we do, we fall even further behind… That's what brings us stress”.*
	Being alone in the crowd	48-year-old bus driver, with 24 years of seniority	*“**It feels like we become invisible**. With time, we learn to put things into perspective, but the fact is that so many people pass by us throughout the day, and yet I can’t say I’ve really spoken to anyone. Basically, the driver is just there to listen to complaints…, mainly on bus scheduling”.*
	The toll of an intensive job	41-year-old bus driver, with 8 years of seniority	*“Our job is really intensive, that's just how it is. And this isn’t appealing to anyone. Look, having the salary we get, the level of responsibility, and working from Monday to Sunday with only one rotating day off. **No one wants that these days**… So, saying there's a shortage of drivers is just shifting the problem onto us, when the real issue lies elsewhere”.*
Much more than automation technology	Do not blame the driver	46-year-old bus driver, with 13 years of seniority	*“Here's the thing, they say the service isn’t efficient, buses run late, it's not reliable for passengers. Right, but let me ask: **who***' ***s the first to want the bus running on time? It's the driver, of course!*** *We always want to stick to the timetable, always. We might think it has been badly defined, but we’ll still try our best to follow what's been set”.*
	When automation meets the urban chaos	45-year-old bus driver, with 23 years of seniority	*“As we can see in the video, ABs work well on streets where there is no illegal parking, bus lanes are free, on wide, open streets. I mean, where nothing unexpected happens, no parked car suddenly opens a door, forcing us to divert to the left, for example. Now, let's look at our city, **it***' ***s gotten so many constraints and unexpected situations*** (…) *it*'*s almost as if you’d have to build the infrastructure from scratch”.*
A bus driver behind automation	A rest that could be tiring	51-year-old bus driver, with 24 years of seniority	“[Supervising an AB] *it might seem like a rest, but I think it isn’t quite that. Sure, I’m taking my hands off the wheel, but I’m still on high alert. It*'*s like the kind of rest you get when you’re in the passenger seat and someone else is driving. For us professional drivers, that's not easy. So no**, I wouldn’t call it a rest at all, if anything, the nervous stress can actually increase**. Of course, that might just be in the early stages of this transition to automated transit. Later on, as the machine starts handling the unexpected better, we begin to learn and adapt”.*
	Automating, still driving	47-year-old bus driver, with 23 years of seniority	*“You could say, ‘Well, just put anyone behind the wheel, and if something happens, they’ll react’. But what if that person doesn’t know how to brake properly with a bus full of passengers, on stone paving* [cobbled]*, in the rain? We know how to stop a vehicle this size without braking hard, for instance. **So, will that person know how to do the job when the automated system requires human intervention?**”*

### It is not just about driving

The first theme encapsulates how the drivers stressed that their work activity cannot be reduced to driving in the strict sense. Providing a public service involves offering information and guidance about a complex bus network and the city (e.g., how to get somewhere, or where to change to another bus route), assisting passengers with reduced mobility or older people, ensuring compliance with ticketing rules, and guaranteeing passenger safety and comfort. This dimension has recently been confirmed by other studies, which emphasise that bus drivers are more than operators within a complex urban mobility system; they are agents who assist passengers and use their knowledge of the city to help them navigate it.^14,15^ In this sense, drivers’ activity contributes to more accessible and inclusive mobility, helping to overcome “invisible barriers” within an urban system that is not equally familiar (or intuitive) to all passengers.

The bus drivers described this role as requiring specific knowledge of the city. It is not equivalent to the topographical information readily available through digital navigation tools or even traditional maps. Rather, this knowledge is gradually consolidated through experience and comprises what the drivers described as “the city is in our heads” (see Table 2). This living, experience-based knowledge of the city operates as a multi-layered mental cartography. It identifies roads with critical points (e.g., where delays are likely), narrow streets with sharp turns requiring careful manoeuvring, and possible detours in the event of road closures or obstructions. In particular, when rerouting, a key aspect is balancing an understanding of the city's situational constraints (e.g., road surfaces and their variations under different weather conditions) with the need to maintain service reliability.

“(…) that street is cobbled. So, it's raining, you have to go really slowly, because if something unexpected happens and you brake hard, you’ll never be able to stop the bus. We manage this because we know the city, the places where we can pass, where we have no turning angle, where it gets slippery. It's many days spent in the city” (47-year-old bus driver, with 13 years of seniority).

The “city in the drivers’ heads” is more than a static memorisation of routes; it is a dynamic representation oriented towards the efficiency, safety, and reliability of public transport. Akridge et al.^14^ also described these less visible forms of situated knowledge, noting that they could exceed the capacities of automated systems, since “rerouting requires more than knowing the quickest way around an area” (p. 6).

A similar phenomenon has been documented in the context of metro automation.^48,49^ Karvonen et al.^48^ identified a set of “hidden roles” performed by train drivers, such as knowing how to control the train under different weather conditions, optimising stopping times at stations to keep the service on schedule, or acting as an essential communication node among metro system actors by providing real-time information. The authors note that this expertise is often overlooked in design and planning processes, reflecting an automation-centric approach to system design. In addition, many of these roles (e.g., anticipating unexpected events, responding to environmental variability) are not formally defined tasks but experience-based practices grounded in drivers’ situated awareness and decision-making.^48^ As a result, they remain less visible than the driving task itself, which, because it is routinely performed, may appear deceptively straightforward. These observations provide insights for the design and integration of ABs by highlighting the importance of incorporating workers’ situated knowledge into models of human-automation collaboration. They also support the view that automation should complement, rather than displace, human operators.

### A job no one wants anymore

The bus drivers discussed their working conditions and how these undermine the sustainability of their work. Conditions such as irregular and shift schedules, time pressure, exposure to physical risks (e.g., vibrations, glare from advertising panels or shiny metallic surfaces), repetitive movements (e.g., manoeuvring the bus, opening and closing doors), having to remain in the same position for long periods, and the need to cope with high levels of traffic congestion, were identified as negatively affecting health and well-being. These conditions influence the bus drivers’ perceptions of their ability to continue working in UPPT in the long term. On this point, two factors emerged most prominently in their accounts.

The first issue concerns the insufficient time allocated for bus line routes, including the designated break time, a 10-min period between journeys intended for tasks such as checking the bus, ensuring no passenger belongings were left behind, or using the toilet. Yet, as the drivers explained, “We’re always running late”. The scheduled time for routes is shorter than the actual time needed, given the intense traffic levels (particularly after the pandemic) or unforeseen events such as roadworks. Therefore, break periods are often consumed by delays, leaving no time for standing up or using the toilet.

“If everything goes smoothly, that route takes 40 min. What's in the schedule? 30 min plus 10 of break. But those 10 min are absorbed, because the journey itself takes 40. So, the break time disappears. We arrive at the last stop already late, with no time to pause, and have to turn back immediately. We try, we really do, but the times are calculated as if only buses existed in the city, that's not real life” (42-year-old bus driver, with 21 years of seniority).

These time pressures highlight how current operational and situational constraints may interact with broader demands in future automated UPPT systems. However, poor scheduling has another significant consequence. And this brings us to the second issue: tension and conflict with passengers. Poor scheduling is a major source of friction,^36^ and this occupational risk emerged clearly in the drivers’ accounts.

“We’re talking about all kinds of issues we have to endure. Most of the time, it's about bus schedules. Every passenger wants to wait as little as possible and then get to their destination as quickly as possible. No one understands the setbacks of traffic, the streets, the parking problems, all of it” (48-year-old bus driver, with 27 years of seniority).

The management of these tensions, involving risks of verbal or physical aggression, places the drivers under the need to act upon themselves in order to maintain a certain emotional distance from the conflict, in an effort to control it. Repeated exposure to these situations takes a toll on the health and well-being of drivers. One of the most emblematic examples is the connection the bus drivers made between conflicts and the pain they experience in the neck and shoulder blade area, attributed to the nervous tension of managing confrontations.

Also, tense interactions accentuate a paradoxical aspect of bus drivers’ work, as one driver cogently stated: “The fuller the bus, the more alone I feel” (47-year-old bus driver, with 23 years of seniority). In this way, the bus drivers underscored the solitary nature of their work, despite being surrounded by passengers (see Table 2). The work is experienced as solitary, particularly when managing their difficulties is left to the individual, with situations of tension with passengers standing out as one of the most significant aspects of their work activities.

### Much more than automation technology

While recognising the importance of transitioning towards automated public transport, the drivers emphasised that it cannot be reduced to a purely technological change. In parallel with the promotion of automation as a solution to public transport challenges, a narrative appears to be gaining traction that positions drivers as part of the problem (e.g., delays in bus services or congestion). The bus drivers were unequivocal: “The driver isn’t the problem” (Table 2). For them, this perception stems from the invisibility of their real work activity, which is frequently reduced to the mere act of driving, as we previously addressed (see Section 4.1). Additionally, as higher levels of vehicle automation are expected to take over much of the driving task, and as automation-centred design approaches continue to prevail, bus drivers tend to be relegated to a secondary role in discussions about the future of automated public transport, as if they were onlookers to the transition.

“After all, we’re not just a blank page waiting to be filled. If we’re pushed to the margins, we won’t know what alternatives were on the table, why one path was chosen over another, and in the end, we’ll have had no chance to contribute to the future of our profession” (51-year-old bus driver, with 24 years of seniority).

In this way, the drivers echoed that this transition “is not just about getting technology into buses”. This position does not reflect any form of resistance to change or a lack of technological acceptance. On the contrary, it highlights the importance of integrating current work activities into discussions on automated transport from the earliest stages of design. Rather than advocating for automation at any cost, they argued for the need to closely attend to operational and functional aspects related to work organisation. More concretely, bus route scheduling is a pressing issue today (see Section 4.2) and is likely to become even more critical under the lower operating speeds expected from ABs.

Beyond scheduling, the drivers also stressed the need to “rethink the city itself”, a point that emerged particularly clearly during the third session, when the video was used. Taking the relatively “ideal” scenario of AB operation depicted in the video as a point of comparison, the drivers pointed to multiple urban constraints and sources of unpredictability that characterise their current work, such as illegal parking that hinders bus circulation, the occupation of bus stops, unauthorised use of dedicated bus lanes, and roadworks or closures. These urban factors currently challenge the drivers’ activity and decisively contribute to the perception of inefficiency in bus transport. Within this context of unpredictability, the bus drivers raised concerns about automated public transport, which relies on predictable scenarios that are rarely, if ever, encountered in their daily work.

“It's possible to have more efficient transport, arriving on time and with less queuing to reduce pollution. That's possible, but only if we look at the city itself. Ours has its specificities, I mean, there’re many cobblestone streets, which cause vibrations and damage any technological system. That happened with our automatic ticketing system (…). And then, the city has very narrow streets, so anything can cause a traffic jam, double parking or blocked bus lanes. Driving here takes a lot, getting a bus through spaces you’d never think it could fit” (53-year-old bus driver, with 26 years of seniority).

Indeed, urban constraints may prove problematic for AB operation. Even when not completely blocking the road, badly parked cars interfered with AB circulation and triggered unplanned stops, as sensors detected them as obstacles, requiring human intervention.^50^

Taken together, our findings suggest that planning and designing automated public transport systems involves far more than embedding automation within buses. Participatory design processes for UPPT systems with ABs are thus essential to promote safety, reliability, and improved working conditions. Although ergonomic literature has long stressed the importance of involving future users in the design and implementation of new technological artefacts,^21,23^ worker participation is often restricted to later stages, when the technology has already been designed and introduced into the workplace.^29^ Yet when workers are involved from the outset, participatory design discussions are more likely to move beyond conceptual and technical issues and to address operational and functional constraints. Accordingly, such processes concern less the design of the technology itself than the design of the sustainability of the system of which that technology will form a part.^20^

### A bus driver behind automation

This theme conveys the bus drivers’ perspectives on future work scenarios with ABs and brings to light a tension between technological promises – often achievable in controlled environments – and urban contexts, whose contingencies demand arbitration through drivers’ work activity.

The automation of driving changes the nature of the driving task and reconfigures the driver's role, shifting towards system supervision. In fact, at intermediate levels of automation, drivers are expected to supervise the system and regain manual control following a takeover request (e.g., in the event of unexpected situations, failures, or conditions that exceed the operational design domain of ABs). Yet this supervisory role could be challenging, as the bus drivers reflected (see Table 2). Key here is that supervisory work may increase mental workload, as flagged in the literature.^37,38,51,52,59^ In an empirical study, Monéger et al.^19^ documented tensions experienced by automated shuttle supervisors, especially when systems failed to detect obstacles. Bus drivers, thus, may face a form of machine supervision that is anything but neutral; it requires diffuse attention and may cause tension, particularly under uncertain conditions.

While the physical strain of driving may be reduced, mental and emotional demands may persist, as the human operator becomes the “bodyguard of the machine” (fallback operator). In this sense, and somewhat paradoxically, the promised relief from fatigue “may itself be tiring” (see Table 2), as the bus drivers neatly pointed out. In aviation and, more recently, in the railway sector, this paradox has been recognised: automation can simultaneously reduce workload during already low-workload periods (e.g., during predictable tasks) and sharply increase it in critical or unforeseen situations, due to the need for rapid re-engagement and complex decision-making.^53^ In aviation, for example, this phenomenon has been described as a form of “clumsy automation”.^54^

Despite these challenges, the drivers believed automation would not erase the specificity of their work experience. To substantiate this view, they recalled the continued importance of their expertise in navigating urban variability.

“‘Well, just put anyone behind the wheel, and if something happens, they’ll react’. But what if that person doesn’t know how to brake properly with a bus full of passengers, on stone paving, in the rain?” (47-year-old bus driver, with 23 years of seniority).

In prospecting their work with ABs, the drivers considered that their expertise would not simply vanish with automation. Even with higher levels of automated driving, the urban environment continues to demand practical know-how, not merely legal compliance. Further, the drivers drew analogies to railway and civil aviation automation, where operational environments are much more controlled, but “it's not just anyone who can drive”.

“The thing with aeroplanes shows something else, that automation can pretty much do everything, but the pilots are still there, right? It's not just anyone who can step in (…). That's why I reckon, when it comes to ABs, you need a driver, because if something complicated happens, you’ll have the driving experience. You know, we often say that we’ve got a kind of memory from driving, a set of reflexes, or something. So, it just kicks in” (45-year-old bus driver, with 23 years of seniority).

This skilled action the drivers invoked is not exactly a new “technological skill”; it is an experiential and deeply situated form of know-how, but also a relational one. It is knowing how to navigate streets with sharp corners, react to unexpected situations under adverse weather conditions, manage tense interactions with the public, and assist more vulnerable passengers. These skills extend beyond supervising the vehicle's automated operation; they form the foundation for safer, more efficient, and more accessible public transportation.

## Implications for the sustainability of work in automated public transport

### Synthesis and integration of results

In line with recent research seeking to counter the limited voice that bus drivers typically have in projects aimed at transforming public transport, our study sheds light on the expertise of bus drivers. This is particularly relevant in automation initiatives, where drivers’ work is frequently fragmented into discrete tasks, and interest is primarily focused on the technological requirements for AB operations. Such technocentric framings risk obscuring the organisational level of drivers’ work and how it shapes work performance and occupational risks.^12,18^ Distancing automation debates from drivers’ work experience leads to an “overly rationalised understanding”^14^ of the real circumstances they have to deal with.

By bringing their working conditions to the centre of the discussion sessions, the drivers challenged the representation that positions them as the source of structural inefficiencies in current UPPT. Instead, they drew attention to work organisation, scheduling practices, and the contingencies of the urban road environment. These constraints place drivers in situations that may prevent them from applying their own quality-of-work criteria, grounded in work experience, in order to achieve work well done.^17,22^ Under such circumstances, what Clot^17^ termed “thwarted quality” emerges, that is, situations in which drivers’ agency to perform good-quality work is curtailed, as their activity becomes increasingly governed by a logic of service alone (e.g., compliance with schedules). Although thwarted quality in work performance may repress “the desire for work well done”, it does not make it disappear.^22^ ^(p.44)^ This was particularly visible when the drivers reflected on their future work in the context of AB implementation. Far from resisting automation or workplace innovation, they made clear that this debate is about “more than automation technology”, expressing openness towards technologies that could enhance both their well-being and passenger safety.

Looking ahead, the drivers also debated the potential impacts of automation on their well-being. By prospecting their future activity with ABs, they anticipated that managing uncertainty in complex urban environments could increase stress and fatigue. This reflects concerns in the literature regarding potential work intensification (e.g., when drivers take over from automation in the most challenging situations),^51^ reduced control over work, and the consequent deterioration of job quality.^30,38,40^ The European Transport Workers’ Federation^40^ points towards occupational risks that may take new configurations with automation, including both physical and psychosocial risks (e.g., increased pressure, repetitive tasks, or diminished autonomy).

Furthermore, the bus drivers argued that automation would not erase the specificity of their expertise, particularly in complex urban environments where their skills are critical for ensuring safety and efficiency. They maintained that those supervising ABs must necessarily retain the specificity of driving experience in public transport. On this, Calvert et al.^52^ precisely highlighted the importance of prior work experience, remarking that “if using a public road, a person-in-charge should have both the driving licence but also several years’ experience of driving that class of vehicle” (p. 6). The authors noted that training and retraining processes for supervising automated vehicles should build upon existing driving skills, including understanding vehicle control, knowledge of the environment (e.g., to anticipate urban challenges), and managing attention during automated operations.

### Towards a worker-led just transition

The findings of our study reveal that the pursuit of sustainable mobility is not solely an economic or environmental matter (e.g., optimising journeys or energy use); it also concerns work and its conditions and organisation. Achieving sustainable transport is consequently inseparable from ensuring decent work, thereby making the transition socially sustainable. In this context, there is growing consensus that frameworks for managing just transitions should adopt a worker-led orientation: a transition guided by worker experience, criteria defining work well done and the local and organisational conditions that shape professional activities.^55–57^

Indeed, securing workers’ participation is crucial for designing more sustainable work trajectories, a point long addressed within activity ergonomics and work psychology.^21,22,24,29^ As Bonnemain^58^ recently systematised, failing to take workers’ perspectives into account during transformations of their work gives rise to what is known as “futile voice”, which negatively affects workers’ health. Therefore, managing transitions in a way that empowers workers to engage in discussions with management and designers about divergent and even contradictory criteria concerning quality of work, as well as possible and desirable developments in their jobs with technology, is decisive in making work socially sustainable.^58^

This point can be further understood by looking at the tension, evident in the drivers’ accounts, between a logic of service and a logic of care. On the one hand, organisational criteria on which management bases its decisions emphasise transport efficiency, punctuality, and service continuity; on the other hand, the bus drivers remain committed to passenger safety, comfort, and assistance. From this perspective, work sustainability is also shaped by the extent to which bus drivers are able to perform what, in their eyes, is work well done. When organisational arrangements, such as tight scheduling or inadequate operating conditions, prevent this, the possibility of work well done becomes thwarted, with consequences not only for service delivery but also for workers’ health and well-being.^22,58^ As such, a worker-led just transition is not only about involving drivers in technological change, but also about creating conditions for a “deliberated activity”,^22^ in which the criteria defining quality of work can be collectively debated and reassessed.

In this respect, worker-led approaches, promoted by academic research,^32,61^ international organisations,^37,55^ and Transport Workers’ Federations,^40,56,57^ accentuate the importance of social dialogue, deliberation, and worker participation. By placing drivers’ experience at the centre,^56^ such processes should seek to bring together all relevant actors in the sector (e.g., employers, workers and their representatives, social partners, technical designers and manufacturers of ABs, urban planning stakeholders), in order to secure a just transition to automation for transport workers. Our study helps unpack concrete forms of driver participation in these collaborative and participatory processes:

- Participating in the identification of work-related constraints that automation technology should address.
- Anticipating potential limitations and resources that ABs may introduce into work performance, i.e., the situated acceptance of technology.^27,59^
- Informing the design of training/retraining programmes, ensuring they build upon drivers’ existing skills rather than imposing extrinsic, generic instruction.
- Providing continuous feedback throughout the entire transition, from early discussions to the implementation of ABs in UPPT systems. This feedback should reflect the diversity of the workforce (in terms of age, seniority, and gender).
- Contributing to the integration of feedback into system, operational, and organisational design. For example, in railway automation, Song et al.^49^ put forward the relevance of drivers contributing to setting safety management standards for future system operation.

From this perspective, it is possible to identify key dimensions that should become objects of deliberation in the transition process. These include, for example, how route scheduling should be rethought under automated operations, how responsibilities and tasks should be allocated between drivers and automated systems, what operational leeway should be preserved to support safe and effective work, and how training and retraining should be grounded in drivers’ existing expertise. In this way, our findings move beyond diagnosis by indicating areas in which future work organisation should be collectively discussed.

### Limitations of the study

Our results confirm the relevance of an activity-centred approach, thereby broadening discussions on automated mobility, which often lean towards technocentrism or “sociocentrism”. Nonetheless, we recognise that, for these results to realise their practical potential, they must be fed back not only to the company's decision-makers, but also to political and regulatory stakeholders (e.g., those involved in labour policy and urban planning). This constitutes a limitation of our study. Although the participating bus drivers validated the results, they must be discussed with policymakers, regulators, and bus companies. For instance, one pressing policy implication raised by the drivers’ views concerns the need to adequately prepare urban infrastructure for the operation of automated public transport, since a well-developed infrastructure could enhance the technical reliability of AB systems. Hence, future developments of our research could involve close engagement with designers,^59^ vehicle manufacturers, bus companies, and actors responsible for infrastructure and spatial/urban planning, to reflect on the most appropriate options for developing automated public transport (e.g., dedicated fixed routes or flexible-line operations), considering their potential implications for bus drivers.

Another limitation relates to the study design, which relied exclusively on collective discussion sessions with the drivers. Direct observations were not feasible under the field access conditions agreed with the company. It is important to acknowledge that conducting in situ observations could have enhanced our understanding of the reality of work and helped us map the situated nature of drivers’ experience more precisely. In this respect, complementary indirect methods such as video-based auto-confrontation,^44^ a technique based on the delayed verbalisation of activity, might also have been valuable in deepening the analysis, as drivers could have reflected on their actions and made their work strategies more explicit. This technique could have constituted an initial stage of the discussion sessions, supporting a framework that could strengthen drivers’ power to take part in collective deliberation with others (e.g., decision-makers and designers) to influence their work and its organisation.

Moreover, the inclusion of female bus drivers would have enabled a more nuanced interpretation of the results. On this, Cunha et al.^60^ drew attention to the fact that, while traditionally male-dominated, the bus driving profession has undergone a process of feminisation. The authors demonstrated that, when a gender lens is applied, certain constraints of the profession are particularly unfavourable for women, notably the higher prevalence of irregular working hours and fewer opportunities for career development.

Lastly, it is worth mentioning that our findings are highly context-sensitive, since they are deeply rooted in the organisational and urban context in which the research was conducted. Even so, this does not preclude their potential transferability. The localisation of the knowledge generated, reflecting the specificities of the participating transport company, the city's infrastructure, and the current European legislative framework, may provide conceptual and methodological insights applicable to other settings and automation projects, provided that contextual differences are carefully considered.

## Conclusions

Today, the transport sector appears to be defined by two opposing realities. On the one hand, it is characterised by demanding working conditions, with undeniable impacts on drivers’ health and well-being, leading to growing labour shortages and thereby threatening the viability of public transport. On the other hand, substantial investment in driving automation is reflected in the increasing number of pilot projects and initiatives currently testing the feasibility of ABs in UPPT systems. Automation is presented as a leading solution to many of the sector's difficulties, although it may also entail a reduction in the number of jobs directly linked to professional driving. The challenge, therefore, lies in reconciling these two realities, rather than treating them separately. By ensuring that bus drivers’ work remains central to the planning and implementation of automated public transport, the technological promises associated with ABs can be translated into sustainable outcomes for both the bus driver workforce and the public who rely on these services.

## Notes

a. Automation in buses can be understood across five levels of automation:^42^ levels 1–3 involve situations in which the driver remains at the wheel and is required to intervene, although assisted by automated driving systems; at level 4 (more common in automated shuttles for UPPT), the driver is no longer responsible for actively driving, but supervises the vehicle and intervenes only in situations that exceed the system's automation capabilities; and level 5 refers to full automation, with no human involvement, though this level remains contingent on overcoming significant technical challenges.

b. The video was selected as the featured AB closely resembles the conventional buses currently operated by the drivers (e.g., in terms of length, speed, or passenger capacity).
